# High Prevalence of Vitamin D Deficiency in Critically Ill Patients in Tropical Southern Taiwan: Risk Factors and Clinical Outcomes

**DOI:** 10.3390/jcm15166158

**Published:** 2026-08-07

**Authors:** Shoa-Lin Lin, Jia-Ying Hu, Wei-Cheng Lin, Jun-Hao Wei, Chi-Li Lee, Chih-Neng Hsu

**Affiliations:** 1 Intensive Care Unit, Department of Internal Medicine, Kaohsiung Yuan’s General Hospital, No. 162, Cheng-Kung First Road, Lingya District, Kaohsiung 802635, Taiwan; y6448@yuanhosp.com.tw (C.-L.L.); y3090@yuanhosp.com.tw (C.-N.H.); 2 Department of Nurse Practitioners, Kaohsiung Yuan’s General Hospital, Kaohsiung 802635, Taiwan; 3 Pingtung Christian Hospital, Pingtung 900059, Taiwan; 4 Department of Nursing, Fooyin University, Kaohsiung 831301, Taiwan; aa665@fy.edu.tw; 5 Division of Cardiology, Department of Internal Medicine, Kaohsiung Yuan’s General Hospital, Kaohsiung 802635, Taiwan; y6878@yuanhosp.com.tw (W.-C.L.); y7016@yuanhosp.com.tw (J.-H.W.)

**Keywords:** intensive care unit, mortality, prevalence, risk factor, Taiwan, Vitamin D deficiency

## Abstract

**Objective:** Vitamin D deficiency is a global concern, but its prevalence in critically ill patients in high-sunlight tropical regions is limited. This study evaluated the prevalence of vitamin D deficiency in intensive care unit (ICU) patients in southern Taiwan and identified which variables correlate with deficiency and in-hospital mortality. **Methods:** We prospectively enrolled 221 critically ill patients, categorizing them by serum 25(OH)D levels as follows: Group A (sufficient, ≥30 ng/mL), Group B (insufficient, 20.0–29.9 ng/mL), and Group C (deficient, ≤19.9 ng/mL). Clinical variables, laboratory parameters, and outcomes were analyzed. **Results**: Despite the tropical climate, 33.5% of patients were vitamin D-deficient, and 47.1% were insufficient. Group C patients were significantly younger than other groups (*p* = 0.049). Significant differences between Groups C and A were observed in ICU length of stay (*p* = 0.036) and total hospital stay (*p* = 0.004). Multivariable analysis confirmed that only younger age (OR 0.968, *p* = 0.008) and low albumin level (OR 0.171, *p* < 0.001) were independently associated with vitamin D deficiency. Only serum albumin (*p* = 0.001) and C-reactive protein (CRP) levels (*p* = 0.015) were significantly associated with in-hospital mortality. **Conclusions:** Vitamin D deficiency is highly prevalent among critically ill patients in southern Taiwan, challenging the assumption that tropical ultraviolet exposure is naturally protective. Younger age and hypoalbuminemia are independent predictors of vitamin D deficiency. However, vitamin D levels were not independently associated with in-hospital mortality, which was instead predicted by albumin and CRP levels. These findings highlight a high rate of hypovitaminosis D in ICU but do not support its role as an independent predictor of mortality.

## 1. Introduction

A large meta-analysis of community-dwelling adults indicated that the lowest quintile of 25-hydroxyvitamin D levels was associated with increased all-cause mortality (pooled risk ratio of 1.57; 95% CI 1.36 to 1.81) when comparing with the highest quintile [1]. Low vitamin D status has been linked to various clinical conditions, including certain malignancies such as colon, breast, ovarian, prostate, and lymphoma. Several studies have reported an elevated mortality risk in vitamin D-deficient individuals with these cancers [2,3,4]. Furthermore, other reports also found that patients with severe serum 25-hydroxyvitamin D [25(OH)D] deficiency were associated with increased mortality in non-cancer critically ill patients [5,6].

Despite its clinical importance, vitamin D deficiency remains an overlooked global health challenge, affecting approximately one billion adults worldwide depending on the definitions used [7,8]. Generally, levels ≥30 ng/mL are considered sufficient, 20.0–29.9 ng/mL insufficient, and ≤19.9 ng/mL deficient [9]. The vitamin D status of critically ill patients in Taiwan has not been adequately characterized. We hypothesized that patients admitted to our intensive care unit (ICU) would exhibit a high prevalence of vitamin D deficiency [or low serum 25(OH)D levels].

While the Acute Physiology and Chronic Health Evaluation II (APACHE II) score is a widely accepted measure of illness severity and mortality risk [10,11,12]. Its relationship with vitamin D status in this population warrants further investigation. Therefore, this study aims to: (1) determine the prevalence of vitamin D deficiency among ICU patients in southern Taiwan and (2) identify clinical variables, including APACHE II scores, that correlate with vitamin D deficiency and in-hospital mortality. In other words, this study tried to assess whether vitamin D-deficient patients might have the worst clinical and laboratory parameter (primary outcome) and highest mortality rate (secondary outcome).

## 2. Materials and Methods

### 2.1. Study Patients

Patients aged 20 years or older admitted to our ICU with an expected stay of more than 48 h were enrolled in this study. Patients were divided into 3 groups according to their serum vitamin D [or 25(OH)D] levels as follows: Group A with 25(OH)D level ≥ 30 ng/mL (defined as sufficient group), Group B with 25(OH)D level from 20.0 to 29.9 ng/mL (defined as insufficient group), and Group C with 25(OH)D level ≤ 19.9 ng/mL (defined as deficient group). Group A had 43 patients aged from 38 to 93 (74.16 ± 12.16) years, 32 male and 11 female, Group B had 104 patients aged 20–92 (70.51 ± 14.40) years, 60 male and 44 female, and Group C consisted of 74 patients aged 23–92 (67.47 ± 15.05) years, with 39 male and 35 female. No enrolled patients were subsequently excluded after initial recruitment. The inclusion of patients was slightly unevenly distributed across seasons as follows: spring (34.8%), summer (27.1%), autumn (22.6%), and winter (15.4%). Season of admission was not significantly correlated with serum 25(OH)D levels (*p* = 0.061).

### 2.2. Exclusion Criteria

Patients meeting any of the following criteria were excluded from the study: severely impaired gastrointestinal function, pregnancy or lactation, hypercalcemia (total calcium > 10.6 mg/dL), or tuberculosis.

### 2.3. Laboratory Studies

Blood samples were obtained after an overnight fast, and the following parameters were measured: complete blood cell count, liver and renal biochemistry, lipid profiles, fasting glucose, hemoglobin A1c (HbA1c), intact parathyroid hormone (iPTH), and total 25(OH)D (vitamin D) levels. In other words, data were obtained within 24 h of admission. Additionally, plasma levels of blood urea nitrogen (BUN), creatinine, estimated glomerular filtration rate (eGFR), hemoglobin, albumin, C-reactive protein (CRP), calcium, phosphate, alkaline phosphatase, intact parathyroid hormone (iPTH), hemoglobin A1c, and cholesterol were analyzed in our central laboratory. Serum 25(OH)-D3 levels were measured using an electrochemiluminescence immunoassay.

Chronic kidney disease (CKD) is defined as abnormalities of kidney structure or function present for >3 months. According to the KDIGO 2024 CKD Guideline, CKD is diagnosed by markers of kidney damage (like albuminuria) or an estimated glomerular filtration rate (eGFR) < 60 mL/min/1.73 m^2^ [13]. Acute renal failure was defined as an increase in serum creatinine (Cr) > 0.5 mg/dL if baseline Cr < 3.0 mg/dL, or an increase in serum Cr > 1.0 mg/dL if baseline Cr > 3.0 mg/dL within 3 days, or the development of oliguria (urine output < 400 mL/day or <20 mL/h). The APACHE II score was calculated using the worst physiological values recorded during the first 24 h of ICU admission.

### 2.4. Statistical Analysis

All statistical analyses were performed using SPSS version 26 (IBM Corp., Armonk, NY, USA). Discrete variables are presented as counts (percentage), and continuous variables as means ± standard deviations (SDs). Differences in demographic and clinical characteristics between groups were assessed using the chi-squared test for categorical variables and one-way analysis of variance (ANOVA) was performed to compare the differences among groups. The homogeneity of variance was assessed using Levene’s test. If the assumption of homogeneity of variance was met, standard ANOVA was applied, followed by Bonferroni post hoc test for multiple comparisons. If the variances were unequal, Welch’s ANOVA was utilized, and Tamhane’s T2 test was performed for post hoc pairwise comparisons. Logistic regression models were used to estimate the relationship between associated factors and vitamin D deficiency, as well as in-hospital mortality. To ensure a parsimonious model and prevent overfitting, variable selection was based on clinical relevance and univariable screening; only variables demonstrating statistical significance (*p* < 0.05) in the univariable analysis were subsequently entered into the multivariable logistic regression model. Multicollinearity among predictors was evaluated using Variance Inflation Factors (VIFs), with a VIF > 2.5 indicating potential collinearity. Model goodness-of-fit was assessed using the Hosmer–Lemeshow test.

## 3. Results

### 3.1. Prevalence and Clinical Characteristics of Patients in Three Groups

From November 2019 to November 2021, 221 patients were enrolled and categorized into three groups based on their serum 25(OH)D levels. The prevalence of vitamin D deficiency and clinical characteristics of the three groups are presented in Table 1. The overall prevalence of vitamin D insufficiency and deficiency (Groups B and C) was 80.5%, and the prevalence of vitamin D deficiency alone was 33.5%. Patients in Group C (67.47 ± 15.05 years) were younger than those in the other two groups (*p* = 0.049). Only 19.5% of critically ill patients had a vitamin D level > 30 ng/mL (Group A). In the study patients, 24 patients died and 197 patients survived. The vitamin D levels for survival and non-survival patients were 23.9 ± 8.1 ng/mL and 20.6 ± 7.4 ng/mL, respectively. An independent t-test showed no statistically significant difference, though a borderline lower trend was noted in non-survivors (mean difference −3.3 ng/mL, *p* = 0.058). There was no statistically significant difference in mortality among these three groups (*p* = 0.395), though the mortality rate was numerically higher in Group C (14.9%) compared to Group A (9.3%) and Group B (8.7%).

Using the chi-squared test and ANOVA, no significant differences were found in clinical variables such as septic shock, arrhythmia, acute renal failure, infection with fever, ventilator or vasopressor use, and APACHE II score among the three groups. However, significant differences were observed in ICU length of stay (*p* = 0.036), total hospital stay (*p* = 0.004), serum vitamin D levels (*p* < 0.001), albumin concentrations (*p* < 0.001), and total calcium concentrations (*p* < 0.001) among the three groups (Table 1).

**Figure 1:**
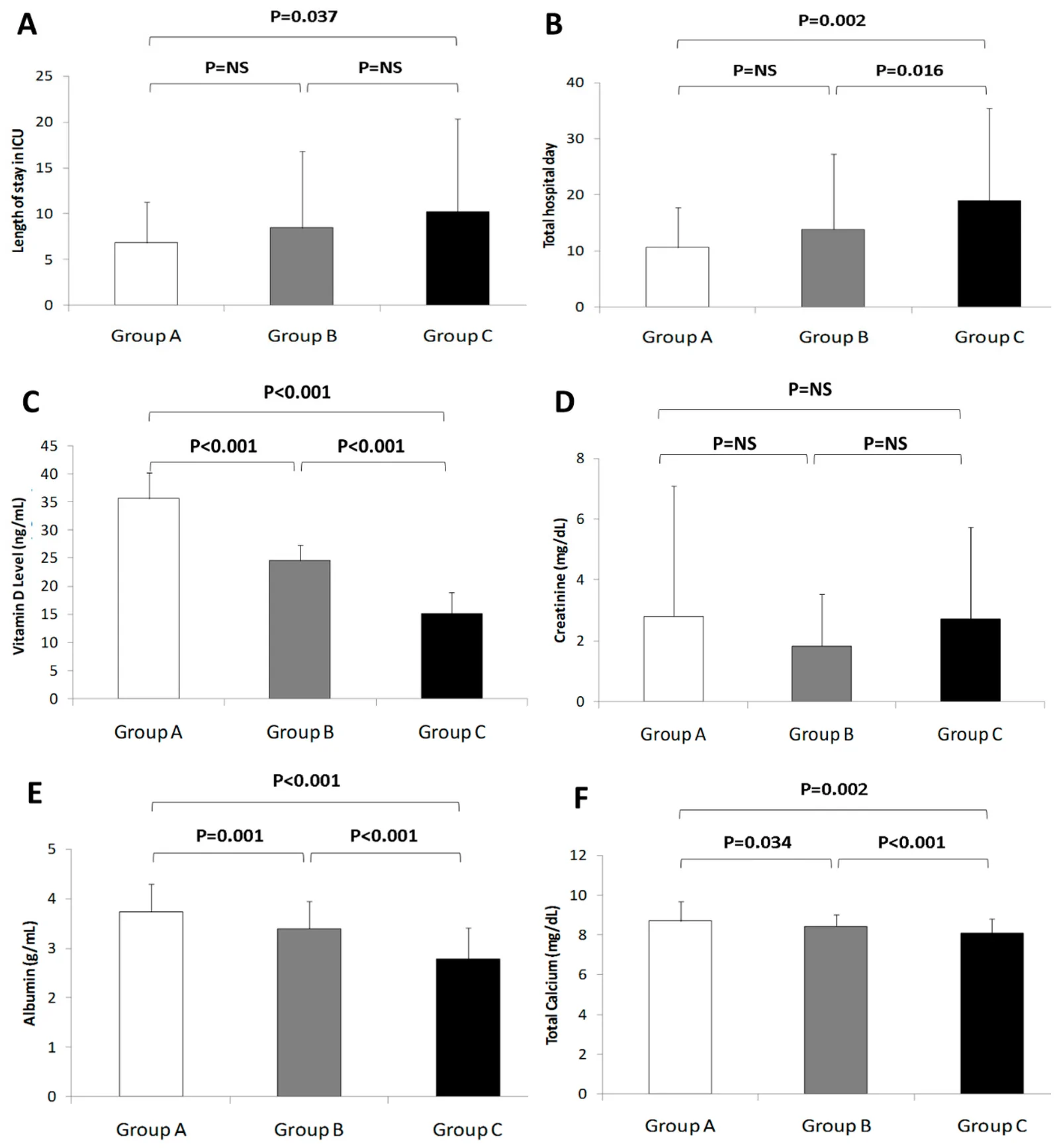
Showing the bar chart of three groups, which demonstrate the inter-group differences in intensive care unit (ICU) length of stay (**A**), total hospital day (**B**), 25(OH)D level (**C**), creatinine (**D**), albumin (**E**), and total calcium (**F**).

Further analysis to determine inter-group differences revealed that the duration of ICU stay (10.24 ± 10.13 days, *p* = 0.040) and total hospital stay (18.96 ± 16.50 days, *p* = 0.001) were longer in Group C patients compared to Group A patients (Figure 1A,B). Vitamin D levels were significantly higher in Group A patients (35.58 ± 4.54 ng/mL) compared to Group B (24.54 ± 2.74, *p* = 0.002) and Group C (15.10 ± 3.76, *p* < 0.001) patients (Figure 1C). Similarly, significant differences in albumin (*p* < 0.001 in all comparisons) and total calcium (*p* < 0.05 in all comparisons) were noted among the three groups (Figure 1E,F). However, no significant differences were found in other biochemistry variables (including BUN, creatinine, eGFR, glucose, HbA1c, phosphate, and intact parathyroid hormone). There was also no significant difference in the ICU admission diagnosis among the three groups (Table 2).

### 3.2. Associated Factors for Vitamin D Deficiency and In-Hospital Mortality

The associated factors for vitamin D deficiency based on univariable and multivariable analyses are shown in Table 3. Univariable analysis identified younger age, longer hospital admission, low albumin levels, and low calcium levels as significant risk factors for vitamin D deficiency. However, multivariable analysis showed that only younger age (*p* = 0.008, OR 0.968, 95% CI 0.946–0.992) and low albumin levels (*p* < 0.001, OR 0.171, 95% CI 0.089–0.328) were significantly associated with vitamin D deficiency. For in-hospital mortality analysis (Table 4), no correlation was found for other clinical factors. However, in both univariable and multivariable analyses, only serum albumin levels (*p* < 0.001, OR 0.159, 95% CI 0.054–0.467) and CRP levels (*p* = 0.015, OR 1.093, 95% CI 1.018–1.175) were significantly associated with in-hospital mortality. After evaluating the event-per-variable (EPV) ratio, we found that the initial statistical method for analyzing the in-hospital mortality has increased the risk of overfitting. We therefore simplified multivariable model by selecting the two primary independent predictors (albumin and CRP) for internal validation using bootstrapping techniques. In Table 4, only statistical data of these two predictors (albumin and CRP) were shown. Thus, in the multivariable models, all VIF values were <2.5. The Hosmer–Lemeshow test indicated good model fit (*p* > 0.05 for both models). 

## 4. Discussion

Our study reveals a high prevalence of vitamin D inadequacy (combined insufficiency and deficiency: 80.5%) among critically ill patients in southern Taiwan. Notably, patients in the deficient group (Group C) were younger and experienced longer ICU and total hospital stays compared to the sufficient group (Group A) (Table 1). Multivariable analysis confirmed that young age and low serum albumin levels were independently associated with vitamin D deficiency, while serum albumin and CRP levels were the only significant predictors of in-hospital mortality (Table 4).

The prevalence of vitamin D deficiency in Western populations may differ from Asian populations. Data on vitamin D deficiency in Taiwan have been limited. A study by Chen KW et al. across four hospitals in northern Taiwan reported that the prevalence of vitamin D deficiency and severe deficiency was 40.5% and 18.2%, respectively, in ICU patients [14]. In their study, patients were divided into four groups according to the 25(OH) D level as follows: sufficiency (>30 ng/mL), insufficiency (20.1–30 ng/mL), deficiency (12.1–20 ng/mL), and severe deficiency (≤12 ng/mL). Therefore, the prevalence of patients with 25(OH) D level < 20 ng/mL was 58.7%. Another community-based cohort study has evaluated the vitamin D deficiency in northern Taiwan. This study divided their population into the following two groups: those with 25(OH)D level <20 ng/mL were considered to be vitamin D-deficient. They found that the 22.4% of the study participants had vitamin D deficiency [15]. In our study, the prevalence of vitamin D deficiency or 25(OH)D level < 20 ng/mL (33.5%) was different from the two reports above. The difference in vitamin D prevalence may be due to different location (the populations of both previous articles live at northern Taiwan), different population and weather (the weather of southern Taiwan was relatively hotter than northern Taiwan).

This study identified a high prevalence of vitamin D inadequacy among critically ill patients in southern Taiwan, a region has a tropical climate characterized by high year-round ultraviolet (UV) exposure. These findings add to the growing discussion surrounding the so-called “tropical paradox”—the notion that living in low-latitude regions with high potential solar radiation automatically prevents hypovitaminosis D, as observed in studies from Malaysia [16] and Indonesia [17]. However, because personal sun exposure, outdoor duration, sunscreen use, clothing habits, and local atmospheric factors were not measured in our study, this interpretation requires caution. While cutaneous UVB synthesis is a primary physiological pathway [18], unmeasured behavioral and environmental factors (such as sun-avoidance behaviors, indoor occupations, air pollution, and skin pigmentation) may contribute to the observed lower vitamin D levels despite the geographical climate.

While 25(OH)D levels can vary seasonally even in low-latitude regions, our analysis showed that the season of admission did not significantly impact vitamin D status. This further supports the “tropical paradox” hypothesis that sun-protective behaviors and indoor lifestyles in southern Taiwan play a more dominant role than chronological seasonality itself. However, because we have not measured individual sun exposure, outdoor activity hours, or lifestyle habits, our explanation attributing the lower vitamin D levels in the relatively younger elderly cohort to “sun-protective behavior and indoor lifestyles” was speculative.

Contrary to the general population where the elderly are often considered to be more prone to have low vitamin D levels, our data showed that the relative younger patients were significantly more vitamin D-deficient. Younger patients in the ICU might often present with acute trauma or sepsis, which can cause a more rapid “acute-phase” drop in vitamin D levels compared to the chronic, age-related decline seen in elderly patients with multiple comorbidities. Our finding was consistent with a previous report from an urban Korean hospital that elder patients also have higher vitamin D levels compared with younger patients in critically ill surgical patients [19].

The strong independent association between low albumin and vitamin D deficiency (*p* < 0.001) observed in this study is consistent with previous research [20]. Albumin is one of the single most useful blood tests to assess nutritional status, as it has a longer half-life than prealbumin (reflecting long-term nutritional status) and is more readily available and reliable [21]. The rate of albumin synthesis may be significantly altered in the critically ill [22]. In the acute-phase response to trauma, inflammation or sepsis, there is a decrease in the synthesis of albumin [23]. Moreover, during the acute phase of critical illness, the dysfunction of the endothelial barrier may cause an increase in the “capillary leakage”. This occurs in patients with sepsis [24] or after major surgical stress [25,26]. The majority of the circulating 25(OH)D is bound to vitamin D-binding protein (VDBP) and albumin [27]. Therefore, the clinical finding of low albumin level may be due to both decrease in the albumin synthesis and the capillary leakage. Meanwhile, since the capillary leak of VDBP and albumin into the interstitial space may cause the decrease in the circulating 25(OH)D, we hypothesize that the low level of vitamin D may also be due to both inadequate nutrition with resultant hypoalbuminemia and the capillary leak of VDBP and albumin.

By multivariable analyses, this study also found that only serum albumin (*p* = 0.001) and CRP levels (*p* = 0.015) were significantly associated with in-hospital mortality. An important review suggests that serum albumin could be an independent predictor of mortality in a wide range of clinical diseases. It reports an estimated increase in the odds of death from 24 to 56% for each 2.5 g/L decrease in serum albumin concentration in patients with acute or chronic diseases [28]. In studies of hospitalized patients, hypoalbuminemia is associated with increased length of stay, higher complication rates and higher mortality [29,30,31]. Large community-based studies have shown a link between low serum albumin and an increase in morbidity and mortality [32,33]. As for the C-reactive protein (CRP), it was a marker of elevated levels of circulating inflammatory and has been widely investigated in many diseases. In a recent community-based prospective cohort study, after adjusting for traditional risk factors, higher baseline CRP levels were significantly associated with increased risk of all-cause mortality, cardiovascular mortality and cancer mortality [34]. Another study also demonstrated a significant direct correlation between CRP levels and six-month all-cause mortality in heart failure patients [35]. Thus, this above information may reasonably explain our findings that hypoalbuminemia and elevated CRP levels were significantly associated with in-hospital mortality.

Our results demonstrated that vitamin D deficiency patients had a prolonged ICU and total hospital stays as well as lower serum albumin level than other two groups (Figure 1), which suggests that vitamin D status is a marker of disease severity. While several review articles and trials suggest potential outcome benefits with daily vitamin D supplementation in patients with severe baseline deficiency [36,37,38,39], our observational data cannot establish a causal link or justify routine screening and supplementation protocols. Instead, these findings highlight the need for future randomized controlled trials to evaluate whether targeted screening and timely replenishment in high-risk ICU populations can meaningfully improve clinical recovery and outcomes.

In order to confirm the multivariable model reliability, the event-per-variable (EPV) ratios of Table 3 and Table 4 were evaluated. For Table 3 data (Vitamin D Deficiency group, *n* = 74 events), a total of four variables (age, total hospital day, albumin, and total calcium) were entered into the multivariable model. This yields an EPV ratio of 18.5 (74/4), which comfortably exceeds the standard statistical recommendation of ≥10. However, for Table 4 data (in-hospital mortality, *n* = 24 events), initially, 11 variables that showed univariate significance were entered into the full exploratory model to avoid missing residual confounding; we acknowledge that this limits EPV ratio (24/11 = 2.18) and increases the potential risk of overfitting. To resolve this weakness, we have performed two major adjustments as follows: firstly, we decrease the study variables in the analysis of multivariable mortality model. We select the two primary independent predictors (albumin and CRP) to satisfy the EPV ≥ 10 rule (24/2 = 12). Secondly, we applied internal validation via bootstrapping techniques to cross-verify the coefficients and ensure the robust reliability of our mortality conclusions. The simplified multivariable model confirms that albumin and CRP remain the only statistically robust independent predictors of in-hospital mortality. We have updated Table 4 to reflect this simplified, statistically sound significant model. (see revised Table 4).

This study has several limitations. First, the number of patients in this study was not sufficient to analyze the effect of vitamin D deficiency for disease-specific subgroup analysis. Second, the outdoor activity level, the environment of sunlight exposure, and some residual confounding factors were not included. In addition, this study might have possible selection bias and was a single center study. Future multicenter studies involve more patients than it may be needed. Third, we did not measure VDBP levels, which might provide a clearer picture of bioavailable vitamin D in the presence of hypoalbuminemia. Fourth, external factors such as pre-admission supplementation or specific indoor-work histories were not captured; further study including more detail external factors may be necessary in the future study. Fifth, our study recruitment period (November 2019 to November 2021) overlapped significantly with the COVID-19 pandemic. Public health restrictions, voluntary isolation, and decreased outdoor activities during this era may have substantially reduced cutaneous vitamin D synthesis due to lack of sunlight exposure. This might be an uncontrolled confounder and an alternative explanation for the high prevalence of vitamin D deficiency. Sixth, given the large number of clinical and laboratory variables analyzed, the risk of type I error due to multiple comparisons cannot be entirely ruled out, although our primary findings regarding length of stay remained significant after stringent post hoc adjustments. Seventh, the formal power calculation was not performed for mortality outcomes. With only 24 cases for the in-hospital deaths, the study was underpowered to definitively evaluate the impact of vitamin D deficiency on mortality. The borderline signal observed in univariable analysis (OR 0.946, 95% CI 0.894–1.002, *p* = 0.060) suggests that a true effect might be needed in future study of a larger, adequately powered cohort. Eighth, due to the relatively low number of deaths (*n* = 24), we simplified our multivariable mortality model to avoid overfitting. While albumin and CRP remained independently associated with mortality in the simplified model, these results should be interpreted with caution, and larger multicenter trials are needed for internal and external validation. Ninth, several important clinical confounders were not fully captured in our dataset, including pre-admission vitamin D supplementation, comprehensive nutritional status assessments, patient BMI, chronic liver disease, and baseline frailty scores. These factors could influence both baseline vitamin D levels and in-hospital mortality and should be systematically tracked in future studies.

## 5. Conclusions

This study demonstrates that vitamin D deficiency is highly prevalent in southern Taiwan’s critically ill patients, independently predicted by younger age and lower albumin level, and correlated with longer ICU stays. However, vitamin D deficiency was not independently associated with mortality. Our data do not support the routine implementation of vitamin D screening to predict or improve mortality outcomes in the ICU. Future prospective, randomized controlled trials are required to determine whether targeted screening of vitamin D level and rapid replenishment protocols in ICU patients can shorten hospital stays and improve clinical recovery.

## Figures and Tables

**Table 1:** Difference in various variables between three groups.

Variables	Group A *n* = 43 (19.5%)	Group B *n* = 104 (47.1%)	Group C *n* = 74 (33.5%)	*p*-Value
Age (yrs)	74.16 ± 12.16	70.51 ± 14.40	67.47 ± 15.05	0.049
Gender—Male (%)	32 (74.4%)	60 (57.7%)	39 (52.7%)	0.063
Ventilator used	13 (31.0)	36 (34.6)	28 (37.8)	0.752
Septic shock	9 (21.4)	29 (27.9)	20 (27.4)	0.709
Use of vasopressor ^1^	12 (28.6)	30 (28.8)	22 (30.1)	0.978
Acute renal failure ^2^	18 (42.9)	36 (35.0)	30 (41.1)	0.579
Infection with fever ^3^	14 (33.3)	35 (33.7)	22 (30.1)	0.877
Arrhythmia	2 (6.1)	6 (5.8)	5 (6.8)	0.964
APACHE II score	21.23 ± 8.73	20.66 ± 9.23	20.72 ± 9.76	0.941
Length of stay in ICU	6.84 ± 4.43	8.44 ± 8.40	10.24 ± 10.13	0.036
Total hospital day	10.65 ± 7.06	13.91 ± 13.37	18.96 ± 16.50	0.004
25(OH)D level (ng/mL)	35.58 ± 4.54	24.54 ± 2.74	15.10 ± 3.76	<0.001
eGFR (mL/min/1.73 m^2^)	46.94 ± 31.66	59.99 ± 36.86	60.22 ± 82.14	0.091
BUN (mg/dL)	41.86 ± 29.55	35.45 ± 29.43	46.42 ± 38.16	0.103
Cr (mg/dL)	2.81 ± 4.29	1.83 ± 1.69	2.70 ± 3.03	0.043
Albumin (g/dL)	3.74 ± 0.56	3.39 ± 0.55	2.79 ± 0.62	<0.001
Total calcium (mg/dL)	8.70 ± 0.98	8.42 ± 0.59	8.07 ± 0.74	<0.001
phosphate (mg/dL)	3.56 ± 1.62	3.63 ± 1.42	3.86 ± 1.78	0.529
Fasting glucose (mg/dL)	185.80 ± 113.80	184.13 ± 100.82	181.22 ± 91.76	0.968
HBA1_C_ (%)	6.57 ± 1.90	6.43 ± 1.51	6.65 ± 1.68	0.674
CRP (mg/dL)	7.31 ± 8.11	7.16 ± 8.71	6.61 ± 7.02	0.872
Intact PTH (pg/mL)	100.68 ± 152.02	86.82 ± 180.78	115.79 ± 123.02	0.500

Data were presented with means ± SD or number (%); Group A, B, C indicate patients with 25(OH)D level ≥ 30 ng/mL, from 20.0 to 29.9 ng/mL (insufficient), or ≤19.9 ng/mL (deficient); ^1^ Vasopressor: either dopamine or norepinephrine; ^2^ acute renal failure: increase in the serum creatinine (Cr) for >0.5 mg/dL if baseline Cr mg/dL < 3.0, or increase in the serum Cr for >1.0 mg/dL for those with baseline Cr > 3.0 mg/dL within 3 days, or developing oliguria (urine output < 400 mL/day or <20 mL/h); ^3^ fever: body temperature > 37.6 °C. Differences between groups were assessed using the chi-squared test for categorical variables and the Welch’s ANOVA for continuous variables. APACHE = Acute Physiology and Chronic Health Evaluation, ICU = intensive care unit, eGFR = estimated glomerular filtration rate, BUN = blood urea nitrogen, Cr = creatinine, HBA1_C_ = hemoglobin A1c, CRP = C-reactive protein, PTH = intact parathyroid hormone.

**Table 2:** Main ICU admission diagnosis of three groups.

Diagnosis	Group A (*n* = 43)	Group B (*n* = 104)	Group C (*n* = 74)	*p*-Value
Acute coronary syndrome (including UA, non-STEMI, and STEMI)	13 (30.2%)	39 (37.5%)	19 (25.6%)	0.239
Heart failure/Ac pulmonary edema (including valvular heart disease, CAD, HCVD, etc.)	13 (30.2%)	16 (15.4%)	9 (12.2%)	0.035
Pneumonia (with septic shock or ac respiratory failure or bronchiectasis with infection)	7 (16.2%)	12 (11.5)	14 (18.9%)	0.381
Arrhythmia (tachycardia, complete AV block, bradycardia with syncope, hemodynamically compromised VT, Sick sinus syndrome, PSVT)	4 (9.3%)	5 (4.8%)	6 (8.1%)	0.528
Acute renal failure on CKD, or CKD with AKI	1 (2.3%)	4 (3.8%)	6 (8.1%)	0.293
Diabetes ketoacidosis, or HHS/HHNK	1 (2.3%)	2 (1.9%)	0	0.457
Pulmonary embolism	1 (2.3%)	6 (5.7%)	0	0.090
CAD, s/p PCI or stenting (with or without cardiogenic shock)	0	3 (2.8%)	4 (5.4%)	0.267
Asthma/COPD with acute resp. failure	1 (2.3%)	5 (4.8%)	2 (2.7%)	0.668
Sepsis or septic shock related to infectious colitis, biliary tract infection, liver abscess, UTI or cancer	1 (2.3%)	3 (2.9%)	7 (9.5%)	0.093
Uremia (or ESRD) with severe hyperkalemia	0	3 (2.8%)	2 (2.7%)	0.537

Data were presented as *n* (%) unless indicated otherwise. Groups A–C: indicate patients with 25(OH)D level ≥ 30 ng/mL, from 20.0 to 29.9 ng/mL, or ≤19.9 ng/mL; UA: unstable angina, non-STEMI: non-ST segment elevation myocardial infarction, STEMI: ST segment elevation myocardial infarction, CAD: coronary artery disease, HCVD: hypertensive cardiovascular disease, VT: ventricular tachycardia, PSVT: paroxysmal supraventricular tachycardia, CKD: chronic kidney disease, AKI: acute kidney injury, HHS: hyperglycemic hyperosmolar state, HHNK: hyperglycemic hyperosmolar nonketotic coma, NTG: nitroglycerin, PCI: percutaneous coronary intervention, COPD: chronic obstructive pulmonary disease, UTI: urinary tract infection, ESRD: end-stage renal disease, UGI: upper gastrointestinal.

**Table 3:** Associated factors for vitamin D deficiency (≤19.9 ng/mL).

Variables	Univariate Analysis			Multivariate Analysis		
	OR	95%CI	*p*	OR	95%CI	*p*
Age (yrs)	0.980	0.961–1.000	0.046	0.968	0.946–0.992	0.008
Gender—Male	0.642	0.364–1.132	0.126			
Ventilator used	1.244	0.695–2.230	0.462			
Septic shock	0.993	0.524–1.881	0.982			
Use of vasopressor	0.996	0.536–1.851	0.999			
Acute renal failure	1.116	0.626–1.989	0.710			
Infection with fever	0.799	0.433–1.473	0.472			
Length of stay in ICU	1.031	0.998–1.064	0.064			
Total hospital day	1.031	1.009–1.053	0.005	1.009	0.986–1.032	0.452
eGFR (mL/min/1.73 m^2^)	1.002	0.997–1.006	0.540			
BUN (mg/dL)	1.007	0.998–1.015	0.119			
Cr (mg/dL)	1.068	0.968–1.178	0.189			
Albumin (g/dL)	0.167	0.095–0.293	<0.001	0.171	0.089–0.328	<0.001
Total calcium (mg/dL)	0.468	0.305–0.718	0.001	0.894	0.543–1.472	0.660
Phosphate (mg/dL)	1.113	0.935–1.326	0.229			
Fasting glucose (mg/dL)	0.999	0.996–1.002	0.489			
HBA1_C_ (%)	1.058	0.896–1.248	0.507			
CRP (mg/dL)	0.985	0.950–1.021	0.416			
Intact PTH (pg/mL)	1.001	0.999–1.003	0.305			
APACHE II score	0.999	0.969–1.030	0.954			

Abbreviations: same as in Table 1.

**Table 4:** Associated factors for in-hospital mortality.

Variables	Univariable			Multivariable				
	OR	95%CI	*p*	β	SE	Adjusted OR	95% CI	*p*
Age (yrs)	1.065	1.021–1.110	0.003					
Gender—Male	0.791	0.338–1.855	0.590					
Ventilator used	12.193	3.991–37.254	<0.001					
Septic shock	7.286	2.919–18.187	<0.001					
Use of vasopressor	7.647	2.989–19.565	<0.001					
Acute renal failure	7.036	2.501–19.793	<0.001					
Infection with fever	6.341	2.491–16.143	<0.001					
Length of stay in ICU	1.088	1.046–1.133	<0.001					
Total hospital day	1.017	0.991–1.043	0.195					
eGFR (mL/min/1.73 m^2^)	1.002	0.995–1.008	0.631					
BUN (mg/dL)	1.015	1.004–1.026	0.008					
Cr (mg/dL)	1.041	0.920–1.178	0.525					
Albumin (g/dL)	0.156	0.071–0.340	<0.001	−1.530	0.424	0.217	0.067–0.478	0.002
Total calcium (mg/dL)	0.626	0.360–1.088	0.097					
Phosphate (mg/dL)	1.130	0.878–1.454	0.342					
Fasting glucose (mg/dL)	1.002	0.998–1.006	0.442					
HBA1_C_ (%)	0.795	0.542–1.168	0.243					
CRP (mg/dL)	1.134	1.079–1.191	<0.001	0.110	0.028	1.116	1.045–1.214	<0.001
Intact PTH (pg/mL)	1.001	0.999–1.003	0.194					
APACHE II score	1.097	1.049–1.147	<0.001					
25(OH)D Level (ng/mL)	0.946	0.894–1.002	0.060					

Abbreviations: same as in Table 1. Please see text for detail.

## Data Availability

The datasets generated and/or analyzed during the current study are not publicly available due to privacy and ethical restrictions but are available on request from the corresponding author.

## Abbreviations

ICU = intensive care unit, ANOVA = one-way analysis of variance, APACHE = Acute Physiology and Chronic Health Evaluation, BUN = blood urea nitrogen, Cr = creatinine, CRP = C reaction protein, eGFR = estimated glomerular filtration rate, HBA1_C_ = hemoglobin A1c, iPTH = intact parathyroid hormone, RCT = randomized controlled trial, VDBP = vitamin D binding protein, UV = ultraviolet.
